# Demonstrating the feasibility of digital health to support pediatric patients in South Africa

**DOI:** 10.1002/epi4.12527

**Published:** 2021-09-02

**Authors:** Elin Haf Davies, Karen Fieggen, Jo Wilmshurst, Obuchinezia Anyanwu, Richard Joseph Burman, Sandra Komarzynski

**Affiliations:** ^1^ Aparito Ltd Wrexham UK; ^2^ Division of Human Genetics Department of Medicine University of Cape Town Observatory South Africa; ^3^ Department of Paediatric Neurology Red Cross War Memorial Children’s Hospital Neuroscience Institute University of Cape Town Cape Town South Africa

**Keywords:** feasibility, mobile health, refractory epilepsy, wearable technology

## Abstract

**Objective:**

Resources for management of epilepsy in Africa are extremely limited reinforcing the need to develop innovative strategies for optimizing care. Studies have shown that the prevalence of epilepsy in low‐ and middle‐income countries is substantially greater than in more resourced countries. The objective of this report was to demonstrate that mobile Health (mHealth) technologies have the potential to improve the management of epilepsy in Africa.

**Methods:**

The feasibility of technology‐based home monitoring was investigated in an observational study of 40 children with refractory epilepsy or epilepsy associated with intellectual disability and/or behavior difficulties in South Africa. Technology‐based home monitoring was implemented for six months. Physical activity, sleep, and heart rate were continuously monitored with a wearable device. Caregivers completed regular mobile Patient Reported Outcomes (mPROs) and reported seizures and ad hoc events using a dedicated app. Feasibility was assessed and descriptively measured for recruitment, retention, and engagement of the participants.

**Results:**

The mHealth technology was able to capture important information that gives an impression of the overall experience of the children and their caregivers. Thirty‐seven participants (94.9%) reported at least one clinical event. Seventy‐nine percent of caregivers reported seizure events in their children, which were the primary event anticipated. Median engagement with the wearable device and monthly mPROs was 30.8% and 57.1%, respectively. However, most participants (87%) had to be given smartphones for them to have Bluetooth capabilities and access to the study app. Tolerability to the device was impacted by the difficult living circumstances of caregivers that induced fear of loss or theft.

**Significance:**

The study showed how the use of remote patient monitoring in the form of mHealth can benefit epilepsy patients, despite highly variable engagement with the technology. The combination of mPROs and wearable devices generated informative datasets that will allow clinicians but also the children and their caregivers to better understand and manage the disease.

**Key Points**

- mHealth technology appears feasible in an epileptic‐specific context within a resource‐limited setting but its utility is limited by local barriers.
- High data can be captured in this setting despite local challenges.
- The mHealth technology enabled clinicians to observe their patients outside of the clinic which may enable further tailored treatment.
- Innovations accounting for socioeconomic factors are needed to optimize impact of mHealth technology.

## 1 INTRODUCTION

Epilepsy is a complex neurological disease which is debilitating and poses a significant burden for carers. International studies report an incidence of 61.4 per 100 000 person‐years,^1^ with an estimated 75% of cases presenting in childhood.^2^ The heterogeneity of seizures can make diagnosis and management challenging. Further infrequent or short seizures may limit hospital‐based assessment to elicit key information.

Antiseizure medications (ASMs) are widely prescribed to manage epileptic seizures and should be influenced by the seizure pattern.^3^ Comorbidities, seizure‐related adverse events, and adverse effects caused by the ASMs can have a negative impact on treatment outcomes and add to the healthcare burden and challenges of delivering care.^4^, ^5^

Epilepsy has a major impact on health and survival in low‐ and middle‐income countries (LMICs) especially, with 80% of epilepsy‐associated deaths occurring in these regions.^6^ Epilepsy poses a significant burden in South Africa as identified by the 2016 study in which it was ranked as the highest for disability adjusted life years in the Southern sub‐Saharan Africa region.^7^ Various factors which may produce poor treatment outcomes are observed in South Africa.^8^ Many personal and cultural beliefs can influence adherence to treatment.^9^, ^10^ Furthermore, an estimated 84% of the population most of whom are low‐income earners or unemployed are reliant on resource‐constrained public healthcare facilities which can result in limited access to optimal resources, particularly for those living beyond the urban metros.^11^

The need for additional methods or frameworks to aid with the management of epilepsy, particularly in LMICs, is evident.^12^, ^13^ Precision medicine initiatives have the potential to improve care in epilepsy. Remote patient monitoring in the form of mHealth, described by the World Health Organization as “use of mobile and wireless technologies to support the achievement of health objectives,”^14^ can provide requisite data sources required to understand the patients' experience. mHealth applications can achieve this through the synthesis of real‐world data repositories and on a continuous basis which will allow clinicians to monitor disease progression, response to treatment, and the impact on quality of life. Through these and similar approaches, an in‐depth patient‐specific profile can be generated to guide treatment regimen and help clinicians and researchers better understand the disease.

Smartphone use is on the rise in sub‐Saharan Africa which provides a sizable and unique opportunity for the implementation of digital health technologies. Within South Africa specifically, 84 of households have a mobile phone,^15^ and 51% of adults own a smartphone, which is the highest proportion in sub‐Saharan Africa.^16^

Precision medicine is often viewed as a tool only viable for highly developed well‐resourced healthcare systems, yet LMICs stand to benefit equally if not more from such strategies. The primary objective of this study was to assess the feasibility of precision medicine initiatives and home‐based mHealth to improve the management of children with epilepsy in South Africa. The secondary objective was to assess the potential effectiveness of mHealth through smartphone apps and wearable devices in supporting families of children living with complex epilepsy in LMICs.

## 2 PATIENTS AND METHODS

### 2.1 Patients and recruitment

Participants were children aged four years and over, managed by the epilepsy service at the Red Cross War Memorial Children's Hospital in Cape Town. Patients were eligible to be included in this study if they had a diagnosis of refractory epilepsy (defined as ongoing seizures despite adequate doses of at least two ASMs) or epilepsy associated with intellectual disability and/or behavior difficulties. Exclusion criteria included children younger than four years of age, children whose caregivers were unable or unwilling to consent, and children for whom assent was appropriate but who were not willing to participate. The study population included children from disadvantaged backgrounds, who were reliant on public health services.

Ethics approval was obtained from the UCT Human Research Ethics Committee (ref 767/2017) prior to commencement of this exploratory prospective study. The study was conducted in‐line with the Declaration of Helsinki.

Informed consent was obtained from the parents or guardians of the eligible children prior to participation. Children older than 8 years of age and with cognitive level of functioning equivalent to this age were invited to give assent to participate in the study. Consent and assent forms were available in the participants' home language.

### 2.2 Study design

For the first three months after recruitment, patients received standard clinical care with paper‐based diary for documenting seizures and clinically relevant events. After that period, a technology‐based home monitoring platform was introduced for a further period of six months. During baseline assessment, the participants completed clinical interviews and self‐report questionnaires including the Child Health Utility 9D (CHU9D),^17^ the EQ‐5D‐Y (child‐friendly version of the EQ‐5D‐3L),^18^ custom medication adherence, ketogenic diet, and sleep questionnaires. All ASMs with their dosage and frequency were established during the baseline visit to provide alert notifications.

**FIGURE 1:**
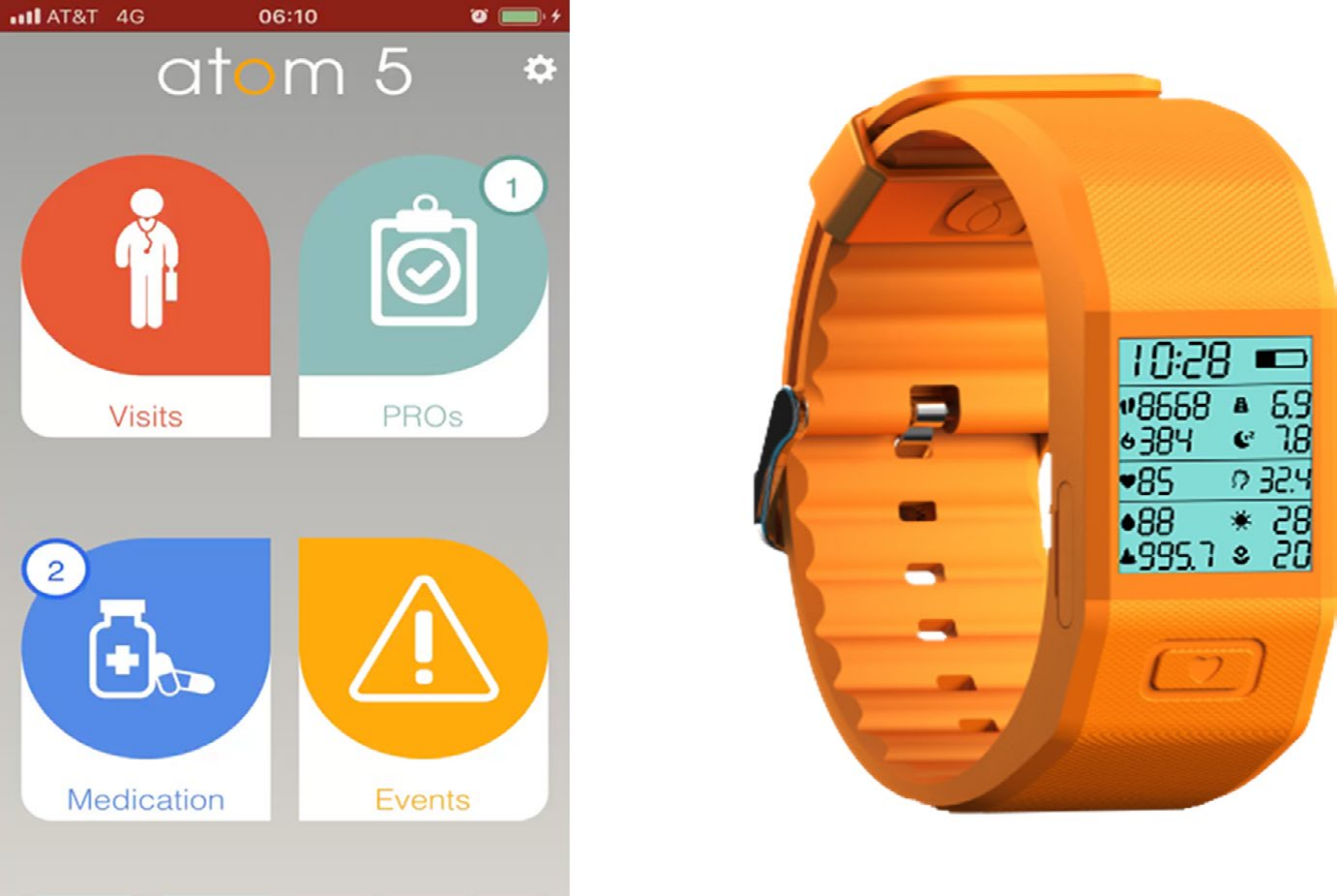
App and wearable device used to collect data

All consenting participants downloaded the Aparito app,^19^, ^20^ via Google or App store (Android and iOS respectively), at the baseline visit. The app was available in English and Afrikaans. The app version used in this study was specifically designed for the study by Aparito and the clinicians from Red Cross War Memorial Children's Hospital. Participants were provided with a smartphone with limited connectivity if their own phone did not meet the technical requirements to allow data transfer and Bluetooth connectivity. The app was paired with a wrist wearable device (Hesvit S3, Hesvit) using Bluetooth (Figure 1). Participants were asked to wear the wrist‐worn device all the time apart from when washing, swimming, and charging the device. The device captured steps, heart rate, and light and deep sleep minutes and calculated sleep duration and type from the 3‐axis acceleration sensor data. Caregivers of the participants were given instructions on how to use the app for reporting their child's seizures (including video capture), behavioral difficulties, poor sleep, illness, or any other events in real‐time. The CHU9D, EQ‐5D‐Y, ketogenic diet, and sleep questionnaire were pushed for completion every 30 days. A yes‐no question was sent through the app every morning allowing the caregivers to report whether their child slept well. The medication adherence report was completed every 90 days. In addition, to support medication adherence reminders were sent via the app at the time the ASM was due. The caregivers were also able to indicate whether the medication was taken and explain the reason why otherwise. Any change in medication prescription was immediately updated on the clinical dashboard to ensure accurate reminders were received by caregivers. A notification was displayed by the app when action was needed. A “Visits” section captured all healthcare appointments. Data were stored on the smartphone and uploaded at predetermined intervals, so uninterrupted Internet access was not required. Cellular data were provided to participants who did not have sufficient resources, and transport costs were refunded for any study‐specific visits to assist with technological difficulties. Participants continued to attend the routine care follow‐up clinic during the study period.

### 2.3 Quantitative data analysis

Feasibility, defined as the ease of implementation of the monitoring technology in the study cohort, was descriptively measured according to recruitment, retention, and participants' engagement. Engagement with the mPROs was computed by dividing the number of actual responses by the number of expected responses over the six‐month study period. Engagement with the wearable device was calculated as the number of days where data were captured for at least four hours in a day, divided by the total number of days in the six‐month period. Days were partitioned at 12PM to avoid subdividing the usual period for sleep. The minimum wear time for engagement computation was set to four hours to include less active patients in the analysis. Engagement with the paper‐based diary recording of seizures gathered in the initial three months was also evaluated. Engagement rates are presented as a percentage.

A qualitative study with a subset of participants to gain a richer insight into these experiences was undertaken and is reported elsewhere.^21^

Descriptive statistics were used to summarize the wearable and mPROs data. Descriptive results are presented as mean and standard deviation for normally distributed data and median and range for not normally distributed data or percentages, as appropriate. Data were analyzed using R software (version 4.0.1).^22^

## 3 RESULTS

### 3.1 Patients

Fifty patients were eligible after screening. Seven could not be contacted, and three declined leaving 40 recruited participants. Within the first two weeks of the study, one participant voluntarily withdrew from the mHealth aspect of the study because they felt overwhelmed by engaging with the devices. This participant's data were excluded from the results presented here.

The analytic sample of mHealth engagement consisted of 19 males (48.7%) and 20 females (51.3%) with a median age of ten years at the time of inclusion, ranging from 4‐16 years. Most of the patients were black African (69.2%). Although more than half were living in formal housing, 69.2% of participants share their bed. Although the majority of caregiver participants (82.1%) were the mother, a number of grandmothers were the primary caregiver (12.8%). Details are presented in Table 1.

**TABLE 1:** Participants' characteristics (N = 39)

Characteristics	Value
Age (years), median (range)	10 (4‐16)
Gender, N (%)	
Male	19 (48.7)
Female	20 (51.3)
Ethnicity, N (%)	
Black African	27 (69.2)
Mixed ancestry	10 (25.6)
European ancestry	1 (2.6)
Indian	1 (2.6)
Type of housing, N (%)	
Formal	26 (66.7)
Informal	13 (33.3)
Bed sharing, N (%)	
Yes	27 (69.2)
No	10 (25.6)
Unknown	2 (5.1)
Primary caregiver, N (%)	
Mother	32 (82.1)
Grandparent	5 (12.8)
Father	1 (2.6)
Foster parent	1 (2.6)

### 3.2 Engagement

#### 3.2.1 Wearable device

One participant's caregiver declined to use the wearable for fear of damage or injury during seizures. Another patient lost the device, leaving a total of 37 participants who provided data with the device. Of 3326 days monitored, 795 days (23.9%) met the definition of a no‐wear day (ie, wear time <4 hours). Of the 37 participants (94.9%) who used the wearables, the median engagement rate was 32.4% (range: 0.5%–98.4%) (Table 2). Missing data occurred when the device was put on charge but was also caused by both wearable and mobile phones being damaged, lost, or stolen. On the days that met the definition of a wear day, the median average time of engagement with the device was 13 hours 56 minutes.

**TABLE 2:** Engagement with wearable device and mPROs

Wearable device (N = 37^a^)	Median (range) average daily wear hours	Median (range) number of wear days	Median (range) engagement rate (%)^b^
Wearable data	13 h 56 min (8 h 44 min‐19 h 58 min)	61 (1‐185)	32.4 (0.5‐98.4)

mPROs (N = 39)	Number of prompts	Median (range) number completed	Median (range) engagement rate (%)
CHU9D	7	4 (1‐7)	57.1 (14.3 ‐ 100)
EQ‐5D‐Y	7	4 (1‐7)	57.1 (14.3 ‐ 100)
Monthly sleep	7	4 (1‐7)	57.1 (14.3 ‐ 100)
Medication adherence	3	2 (1‐3)	66.7 (33.3 ‐ 100)
Daily sleep yes‐no question	188	22 (0‐168)	11.8 (0 ‐ 89.8)

^a^ Two participants did not use the wearable device.

^b^ Engagement rates were computed based on a study duration of 188 days (6 months x 30 days +8 days visit window).

#### 3.2.2 mPROs

All the participants completed the mPROs at least once. Although two participants had been on the ketogenic diet, none were on it during the study. The median engagement with the monthly mPROs was 57.1% (14.3%‐100%), and the median engagement with the medication adherence report was 66.7%. Participants were engaged to a lesser extent with the daily sleep yes‐no question as indicated by a median engagement rate of 11.8% (Table 2).

A prompt with the name and dose of the medication that was required was sent at the prescribed time. As the children received multiple drugs with varying dosage intervals, the number of prompts varied but on average the participants received six prompts per day. Participants' caregivers engaged on average with 55.0% of these prompts to indicate whether their child took the medication. Sixteen caregivers used the prompts to explain why the medication had not been taken. The most prevalent reason was that they forgot but other reasons reported were that the medication was not working, they had none left or that it was making their child ill.

### 3.3 Wearable data

**FIGURE 2:**
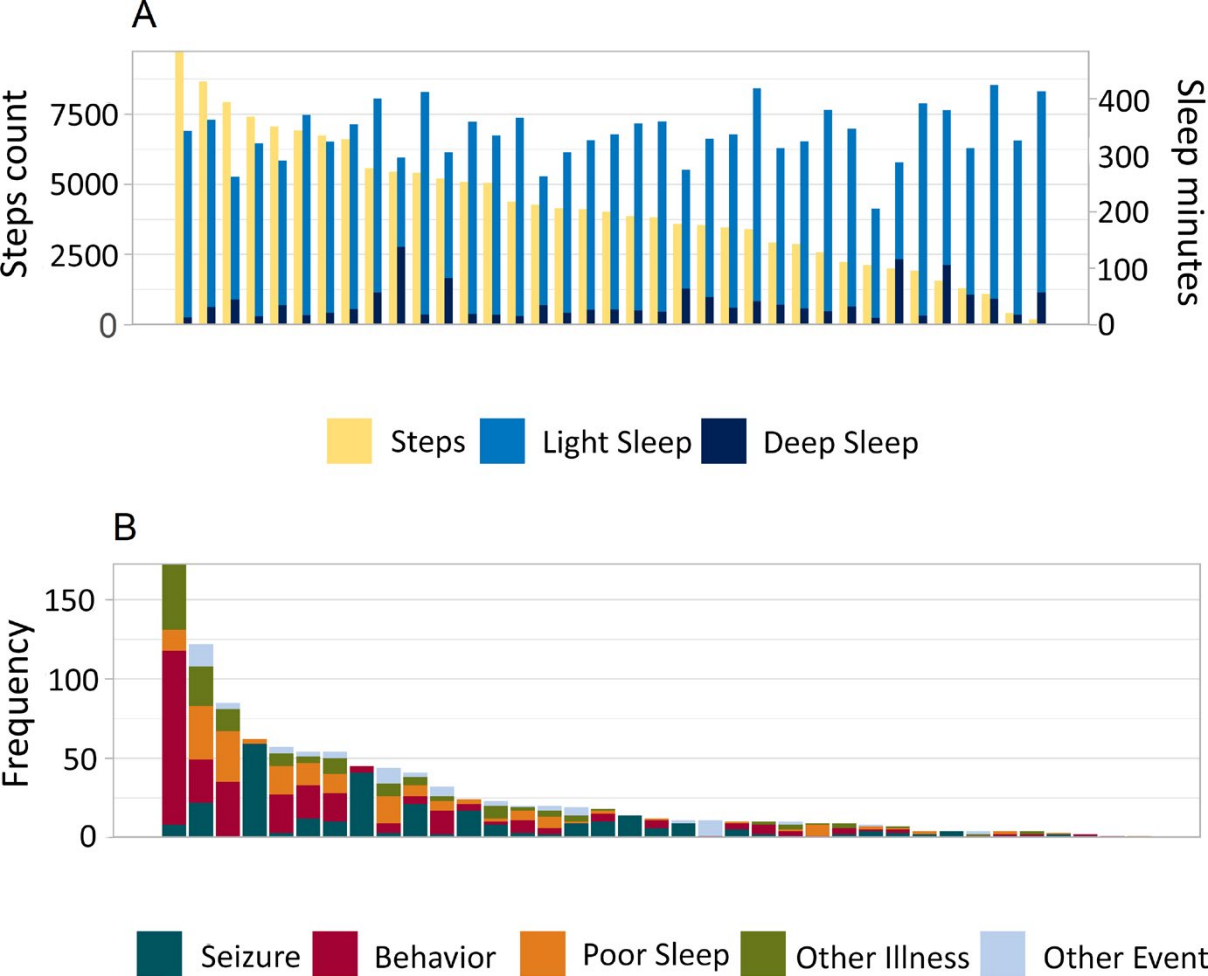
Wearable and events data. A, Average number of steps and sleep minutes recorded by individual participants (N = 37). Each group of bars depicts the data for one patient. B, Number of events reported by individual participants (N = 37). Each bar depicts the data for one patient

The participants were active for a median average of 2 hours 11 minutes (7 minutes‐4 hours 5 minutes) per day in which they did a median average of 4252 steps (189‐10 363 steps). Over the six‐month period, the recorded median average daily sleep duration was 5 hours 58 minutes ranging from 3 hours 15 minutes to 7 hours 55 minutes. The median average proportion of light and deep sleep was 91.7% and 8.3%, respectively (Figure 2A). Heart rate was not consistently recorded by the wearable device and therefore not reported in this paper.

As the aim with this paper is to demonstrate the feasibility of the mHealth monitoring and provide a picture of the data that we were able to capture with the technology, we decided not to report detailed hours and time of recording for the wearable data in this paper. Another manuscript currently in preparation will report the detailed clinical insight gained from the wearable and mPROs data.

### 3.4 mPROs data

For all but one of the nine dimensions of the CHU9D scale, the median average score was less than 0.025 indicating that there were few concerns reported about the quality of life in these participants. The CHU9D scale has a total of 9 domains with an individual domain scoring range of weightings ranging from 0 to 0.1079 when considering all domains collectively. The median average score for the dimension related to schoolwork/homework was 0.05 indicating some concerns. Similarly, the EQ‐5D‐Y scale whose scores range from 1 to 3 where 1 indicates better quality of life found that most of the children had no reported problems, with four out of five quality of life dimensions average scores whose median ranged from 1.0 to 1.6. There were more concerns about the ability to look after themselves as indicated by a median average score of 2.0.

Using the monthly sleep questionnaire, the caregivers reported a median average sleep duration of 8 h for their child (Table S1). Results from the daily sleep yes‐no question completed by the participants showed poor sleep for 23% of the nights.

### 3.5 Clinical events

All participants were given a paper‐based diary in the run up to the study to log their seizures. Only two (5.1%) returned the paper‐based diary at their follow‐up visit which included only a tick to indicate seizure with no further details.

By comparison, 37 of the participants (94.9%) reported at least one clinical event (range: 1‐173). And a total of 287 seizure events were reported by 31 participants (79.5%). The number of seizure events reported by individual participants ranged from one to 59 events. Tonic‐clonic seizure was the most common reported seizure type with 126 (43.9%) events. Tonic and focal unaware seizures were also frequently reported with 84 and 32 events, respectively.

Behavioral issues were reported by 29 participants. A total of 320 events were reported with individual reports ranging from one to 110 events. The most frequently reported behavioral issue was hyperactivity (156 events), and the least reported event was unusual stress level (13 events).

Twenty‐six participants reported 196 poor sleep events (1‐34) via the daily sleep question. The most common reported cause for poor sleep was having trouble falling asleep (77 events), and the least common cause was having nightmares (7 events).

Twenty‐one participants reported 151 other illness events, varying between one and 42 events per child. The most frequently reported event was cough (64 events) while the least reported event was diarrhea (7 events).

Seventeen participants reported 77 “other” events using free text. Caregivers used this field to describe events such as itch or antibiotic use but also to indicate that their child was feeling better (Figure 2B).

Predefined options included in the app for events reporting are detailed in Table 3. Examples of caregivers' comments given using the free text option are included in Table S2.

**TABLE 3:** Predefined options included in the app for reporting events

Event	Predefined options
Seizure	Stiff (tonic); Stiff and jerking (tonic‐clonic); Spasm; Jerk like a shock (myoclonic); Blank (absent); Focal aware; Focal unaware; Details (with possibility to describe in free text)
Behavioral issue	Anxiety; Hyperactivity; Low mood; Social engagement; Attention; Aggression; Unusual stress level; Details (with possibility to describe in free text)
Poor sleep	Pain; Anxiety; Nightmare; Not falling asleep; Frequent wakings; Other (with possibility to describe in free text)
Other illness	Cough; Runny nose; Vomiting; Fever; Diarrhea; Other (with possibility to describe in free text)

### 3.6 Practical, technical, and operational challenges

Most participants (87%) had to be given smartphones with better capabilities upon enrolling into the study. Participants either did not have smartphones or had phones that were not compatible with Bluetooth.

Three phones were lost, three were stolen and ten stopped functioning. Nine participants had handsets repaired or changed, but lost or stolen phones were not replaced. One participant did not report that their phone stopped working.

For the wearables, ten participants reported damage and four loss of the device requiring replacement. All were replaced but breakages and loss would have influenced monitoring.

All participants were sent data monthly at a cost of between R30 and R120 (~US$ 2‐8) a month depending on usage and service provider. A data package of 300MB was sufficient to run the app and upload wearable information for the duration of the study. Only one participant had regular access to Wi‐Fi.

A detailed study cost breakdown is reported in Table 4. Although these costs illustrate cost implications for running the study, they give a useful predictive insight into the overall cost of the intervention for routine health care.

**TABLE 4:** Disseminated project costs

Utility	Cost (R)	Cost (US$)
Prospective costs to the patient		
Data required for mobile app (N = 40)	18 000	1276.74
Phones (N = 22)	35 164.81	2494.24
Phone repairs (N = 9)	680	48.23
Prospective costs to healthcare institutions		
Platform configuration one‐off costs	1 127 872	80 000
Cloud hosting costs	28 196.80	2000
Personnel (year one): students required for study set up, patient recruitment, monitoring support and document translation	159 470.81	11 311.27
Personnel (year two): nursing sister required for patient monitoring and data collection	304 821.23	21 620.98
Wearable devices (N = 39)	27 491.88	1950
Wearable devices replaced (N = 17)	11 983.64	850
Total costs		
Total	1 713 681.17	121 551.46

Note

Exchange rates as of 17 June 2021.

## 4 DISCUSSION

The mHealth technology used over the course of this study was able to capture important information that gives an overall impression of patients’ experiences with complex pediatric epilepsy and also their caregivers. Despite physical activity being beneficial for psychosocial wellbeing, and studies showing its role in seizure mitigation, the majority of epilepsy patients are less active in contrast with the wider population.^23^, ^24^ In addition, seizures have been observed to negatively impact sleep: altering proportions of varying stages and the overall quality.^25^ Consequently, recording the lifestyle parameters of activity levels and sleep longitudinally may be useful in gauging the severity of epilepsy, the efficacy of a prescribed ASM or determining whether holistic prescriptions need to be made to improve disease management and quality of life. Caregiver's reports complemented with the activity and sleep patterns of the child generated by the wearable device provide the clinician with detailed information which should highlight barriers to providing adequate care. Caregivers’ perception of their child's health might be influenced by their difficult living conditions. A discrepancy was observed between the reported sleep duration (8 h) by the caregiver and that measured by the device (5 h 58 min). This could be explained by the improper wearing of the device but also by caregivers not being aware of sleep disturbance. The living circumstances of participants influenced adherence to the wearable, in that many were too nervous or anxious to use the wearable outside of the home. That possibly impacted on the compliance rate and data quality. The use of CHU9D and EQ‐5D‐Y detailed the psychosocial impact of the disease on patients, with median values of 0 for two dimensions of CHU9D and values of less than 0.025 for six dimensions out of nine, and with caregivers for the affected children indicating that they had “no problem” in four out of a total five dimensions. This indicates an optimistic outlook and could influence reporting of events to health providers at clinic visits. Medication adherence was logged using the technology and showed that most caregivers adhere to giving their children's treatment regimen and use the app to report their reasons for not doing so more than when they are in the clinic with doctors. The most common behavioral event recorded was “hyperactivity” which is line with what is known for this group.

Seizures were the primary clinical event as anticipated, with 79% of caregivers reporting its occurrence. Most the children recruited are defined as having refractory epilepsy which would indicate that most of the remaining 21% of the children were likely to have experienced seizures that were not reported. Nocturnal seizures which manifest as bad dreams or absence seizures that present as staring episodes are difficult to detect and thus might have been missed. However, reporting via the app was significantly higher than that achieved on the paper‐based diary (79% vs 5%), demonstrating that the app has a clear benefit in documenting the seizure history of a patient which would have a tangible effect on patient management. While the majority of the children met the definition of refractory epilepsy, in the African setting children can be labeled refractory but in fact may not have had an adequate trial of ASMs due to a lack of sustainable supply and access to ASMs. Some children had apparently controlled seizures with ASMs but significant impact on quality of life from associated comorbidities such as cognitive delay or behavioral issues. As a result, a broader inclusion to the group was undertaken which included established epilepsy but also the presence of comorbidities. Thus, considering the expanded concept of “complex epilepsy” might be needed, especially in the African setting.

In addition, several other events, such as low mood or attention issues, were reported that are not routinely discussed during their hospital/clinic visits. Capturing these data routinely will ensure that all factors which contribute toward the burden of disease across patients are accounted for.

The ability for mHealth technology to aggregate patient physiological, psychosocial, and clinical event data on a longitudinal basis can be of great use to clinicians as this could contribute to the generation of panoramic patient‐specific profiles. As a result, there may be potential for patient treatment and care to be tailored with greater precision according to real‐time seizure frequency reporting.

Our study was limited by the small sample size (N = 40) and thus will benefit from further validations in a larger group of patients. Although the technological feasibility is demonstrated, the context within which this technology was deployed may impact wider use. Median engagement with the wearable and monthly mPROs was 32% and 57%, respectively (Table 2). Tolerability of the wearable was impacted by factors such as behavioral and cognitive events, including intellectual disability and hyperactivity along with the difficult living circumstances of caregivers that induced fear of loss or theft and potential compromise to personal safety in using these devices. Many participants were not familiar with smartphone technology and were unsure of what was required and felt overwhelmed by the instructions given.^21^ Bluetooth connection and upload of data from the wearable were particularly challenging. Limited access to networks, particularly when visiting rural areas as well as inconsistent supply of mobile data, may have also hampered data collection. Lastly, costs incurred must be considered: with an estimated 49% of the adult population in South Africa living below the upper‐bound poverty line (US$ 70.90 monthly income),^26^ full engagement with mHealth technology may not be a viable option with current pricing structures. For instance, the total cost for the smartphones used in the study was US$ 2,494 (US$ 113 per phone) and the data packages provided on a monthly basis were worth between US$ 2 and US$ 8. As a reference, with a figure of 2338MB for average mobile data expenditure per month in South Africa being forecasted for this year from a 2016 report,^27^ it is probable that patients would still require data bundles that exceed 1GB to support additional smartphone utilities, despite a minimum of 300MB being sufficient for use of the mHealth app. The average costs for 1 GB and 2 GB data bundles have been recorded as US$ 9.04 and US$ 14.43, respectively, according to a 2017 report from the Independent Communications Authority of South Africa (ICASA).^28^ Therefore, the cost impact on low‐income patients may not be feasible at all or may be increased beyond affordability if they require a high frequency of engagement with the mHealth app, and/or a particular smartphone.

The costs for running the mHealth platform included a one off set up fee and ongoing cloud hosting costs, this along the wearable devices' costs are detailed in Table 4. The cost of implementation has already been acknowledged as a possible barrier in sub‐Saharan Africa.^14^ With such a barrier in mind, it is important to consider not only the cost to the patient, but also potential costs that may be incurred by healthcare facilities that choose to employ mHealth technologies; this may or may not also lead to cost savings in other areas such as travel, time off work, and waiting times in clinics.

It is imperative that the deployment of digital health solutions is supported in LMICs. With applications within health rapidly becoming digitized, there is a risk that disadvantaged communities may be left behind, which would negatively impact on treatment outcomes and widen the already existing global health inequality gap. Furthermore, as elements of artificial intelligence and machine learning become more commonplace within digital health applications, it is important that data are aggregated from varied patient groups to ensure that no demographic group is ostracized and that all are included in the development of digitized healthcare systems.^29^ Digitized healthcare approaches have been used in sub‐Saharan Africa as early as 2003,^30^ and, within South Africa specifically, prior studies have displayed the acceptability and utility of mHealth for patients showing that there is a digital foundation which can be built upon.^31^, ^32^ That engagement in, and enthusiasm for, digital solutions is greater in young individuals is not surprising but qualitative data from our study show that older people understand the value of mHealth and can be supported to engage.^21^ This is in alignment with recent findings from South Africa which showed a positive correlation between individuals aged between 46 and 60 years who resided within 1 kilometer of their clinic, and an eagerness to engage with a mobile application for the purpose of ordering medication.^33^ Health applications, combined with the present technological momentum, are seeing more people across sub‐Saharan Africa access mobile technology and provide a great opportunity to potentially improve the treatment outcomes of billions of patients.^34^ Additionally, the COVID‐19 pandemic has highlighted the importance of digital healthcare solutions within clinical practice as they are highly adaptable, are efficient, and ensure for the continuation of health care when physically attending an appointment is not advised.^35^, ^36^, ^37^, ^38^ The current international momentum driving the adoption of digitized healthcare solutions is one that may see an increase in use of such technology within sub‐Saharan Africa.

## 5 CONCLUSIONS

Despite variable engagement and individual access to smartphone, the study demonstrated how the use of mHealth can benefit epilepsy patients for remote patient monitoring. The generation of informative datasets will enable all stakeholders including patients and caregivers to better understand and manage their disease. Analysis of qualitative data in conjunction with acknowledging and learning from the experience of patients, clinicians, and carers will allow for a future deployment of technology better suited to the needs of patients and the environment in which it is set. With this proof of concept displayed, and drawing from the lessons learned, extending to a larger group of patients with an app available on more commonly used mobile phones will be required to deliver the aspired impact of this mHealth intervention across all communities globally.

## CONFLICT OF INTEREST

OA and SK are employees at Aparito. EHD is employee at Aparito and holds shares. RJB, KF, and JW have no competing interests.

## ETHICAL APPROVAL

We confirm that we have read the Journal's position on issues involved in ethical publication and affirm that this report is consistent with those guidelines.

## Supporting information

Supplementary Material
