# Supplementary material for: Demonstrating the feasibility of digital health to support pediatric patients in South Africa
Source: Epilepsia Open. 2021 Sep 2;6(4):653–62. doi: 10.1002/epi4.12527 (PMC8633462; doi:10.1002/epi4.12527)
Supplement: Supplementary file 1 — Supplementary Material [file EPI4-6-653-s001.docx]

**Table S1.** Mobile Patient-Reported Outcomes (mPROs) results for the 39 participants.

| **mPROs** | **Dimension** | **Median (range)^a^** |
| --- | --- | --- |
| CHU9D | Worried | 0 (0-0.0227) |
|  | Sad | 0.007 (0-0.0722) |
|  | Pain | 0 (0-0.1426) |
|  | Tired | 0.0160 (0-0.0479) |
|  | Annoyed | 0.0062 (0-0.0313) |
|  | School Work/ Homework (such as reading, writing, doing lessons) | 0.0487 (0-0.0656) |
|  | Sleep | 0.0030 (0-0.0506) |
|  | Daily routine (things like eating, having a bath/shower, getting dressed) | 0.0185 (0-0.0930) |
|  | Able to join in activities (things like playing out with your friends, doing sports, joining in things) | 0.0221 (0-0.1079) |
| EQ-5D-Y | Mobility (walking about) | 1.3 (1.0-3.0) |
|  | Looking after myself | 2.0 (1.0-3.0) |
|  | Doing usual activities (for example, going to school, hobbies, sports, playing, doing things with family or friends) | 1.6 (1.0-3.0) |
|  | Having pain or discomfort | 1.2 (1.0-2.2) |
|  | Feeling worried, sad or unhappy | 1.0 (1.0-3.0) |
| Monthly sleep | Average sleep duration (hours) | 8 (5-10) |

^a^The median (range) score is presented for the CHU9D and EQ-5D-Y mPROs and the median (range) duration is reported for the sleep mPRO.

The Child Health Utility 9D (CHU 9D) (UK weighted tariff) (Stevens K. Qual Life Res Int J Qual Life Asp Treat Care Rehabil. 2009) was developed as a measure of a patient’s health-related quality of life. This PRO has a total of nine domains with domain scores ranging between 0 to 0.1079. A lower score implies less concern with the specific domain.

The EQ-5D-Y (Burström K et al. Acta Paediatr. 2014) and was developed by the EuroQol Group as a generic instrument measuring health-related quality of life in children and adolescents. Each of the five dimensions are scored from 1 to 3 where 1 implies no problems.

**Table S2.** Examples of participants’ free text comments associated with reported clinical events.

| **Clinical event** | **Free text comment examples (non-exhaustive examples).** |
| --- | --- |
| Seizure | “Teacher reports multiple absence seizures.” |
|  | “The seizure was extremely intense and he needed oxygen as his hands and mouth was blue.” |
|  | “Seizure was very intense and after seizure stopped he was very upset and restless as if hallucinating.” |
|  | “3 seizures in one day. I can’t give her the new meds anymore she's smacks herself a lot after the seizures.” |
| Behavioural issue | “Unable to calm down and concentrate on any tasks.” |
|  | “Yesterday morning and afternoon she was not her usual playful self but was easily irritated and crying often with more than usual tantrums.” |
|  | “She's so aggressive and I don't know how to handle her at all.is it the medication or is the brain coz of the illness?” |
|  | “Displayed extreme stubbornness when she had to take her evening dose of tablets. Refused to swallow her tablets and kept it in her mouth. She eventually just swallowed it after we chose to ignore her.” |
| Poor sleep | “Pain in my arms and legs.” |
|  | “Feeling strange.” |
|  | “Closed nose which caused him to end up having fits.” |
|  | “Very hyperactive and laughing for more than 3 hours.” |
| Other illness | “Stomach pains.” |
|  | “Not eating properly.” |
|  | “Complaining of gum aches.” |
|  | “Constipation.” |
| Other event | “Got dizzy and wasn't able to walk.” |
|  | “My child is good.” |
|  | “Low mood” |
|  | “My child was not eating well because he had a lot of phlegm and he lost weight.” |
